# Supplementary material for: Matching degree of embodied carbon trade and value-added trade among Chinese provinces (regions)
Source: PLoS One. 2023 Feb 10;18(2):e0277128. doi: 10.1371/journal.pone.0277128 (PMC9917259; doi:10.1371/journal.pone.0277128)
Supplement: Attachment — Submitted filename: Response.docx [file pone.0277128.s003.docx]

**Response to editor and reviewers**

First of all, I would like to thank the editors and reviewers for pointing out the shortcomings of this article. We have revised the paper according to the reviewers' comments. The revisions are marked in red. The following are the responses to the editors and reviewers' comments:

Journal Requirements:

When submitting your revision, we need you to address these additional requirements.

1. Please ensure that your manuscript meets PLOS ONE's style requirements, including those for file naming. The PLOS ONE style templates can be found at 
https://journals.plos.org/plosone/s/file?id=wjVg/PLOSOne_formatting_sample_main_body.pdf and 
https://journals.plos.org/plosone/s/file?id=ba62/PLOSOne_formatting_sample_title_authors_affiliations.pdf

**Reply:** Thank you for your valuable advice. The manuscript has been modified to meets PLOS ONE's style requirements according to the above links.

2. Please note that PLOS ONE is designed to communicate original research and research methods, and we do not consider policy papers. As such, we ask you to remove policy discussion and recommendations made in your Conclusions section and ensure that statements made in your conclusions are supported by the data in your article.

**Reply:** Thank you for your suggestion. The policy discussion and recommendations in the manuscript has been removed and the conclusions are supported by the data. The corresponding modifications are now included in the revised manuscript.

3. In your Data Availability statement, you have not specified where the minimal data set underlying the results described in your manuscript can be found. PLOS defines a study's minimal data set as the underlying data used to reach the conclusions drawn in the manuscript and any additional data required to replicate the reported study findings in their entirety. All PLOS journals require that the minimal data set be made fully available. For more information about our data policy, please see <http://journals.plos.org/plosone/s/data-availability>.

Upon re-submitting your revised manuscript, please upload your study’s minimal underlying data set as either Supporting Information files or to a stable, public repository and include the relevant URLs, DOIs, or accession numbers within your revised cover letter. For a list of acceptable repositories, please see http://journals.plos.org/plosone/s/data-availability#loc-recommended-repositories. Any potentially identifying patient information must be fully anonymized.

Important: If there are ethical or legal restrictions to sharing your data publicly, please explain these restrictions in detail. Please see our guidelines for more information on what we consider unacceptable restrictions to publicly sharing data: http://journals.plos.org/plosone/s/data-availability#loc-unacceptable-data-access-restrictions. Note that it is not acceptable for the authors to be the sole named individuals responsible for ensuring data access.

We will update your Data Availability statement to reflect the information you provide in your cover letter.

**Reply:** Thank you for your suggestion.Please refer to the following link for the multi-regional input-output table of China in 2012(After registering an account,you can download it for free.)

<https://www.ceads.net.cn/data/input_output_tables?#1089>

Please refer to the following link for the multi-regional input-output table of China in 2015(After registering an account, you can download it for free.)

<https://www.ceads.net.cn/data/input_output_tables?#926>

Please refer to the following link for the carbon emission data of Chinese provinces in 2012(After registering an account, you can download it for free.)

<https://www.ceads.net.cn/data/province/by_sectoral_accounting/Provincial?#970>

Please refer to the following link for the carbon emission data of Chinese provinces in 2015 (After registering an account, you can download it for free.)

https://www.ceads.net.cn/data/province/by_sectoral_accounting/Provincial?#973

The above data has been uploaded as Supporting Information files when submitting the revised manuscript.

4. PLOS requires an ORCID iD for the corresponding author in Editorial Manager on papers submitted after December 6th, 2016. Please ensure that you have an ORCID iD and that it is validated in Editorial Manager. To do this, go to ‘Update my Information’ (in the upper left-hand corner of the main menu), and click on the Fetch/Validate link next to the ORCID field. This will take you to the ORCID site and allow you to create a new iD or authenticate a pre-existing iD in Editorial Manager. Please see the following video for instructions on linking an ORCID iD to your Editorial Manager account: <https://www.youtube.com/watch?v=_xcclfuvtxQ>

**Reply:** Thank you for your suggestion.

Xia Li, the first author of this article, ORCID iD: 0000-0002-3871-061X

Guangyao Deng, the corresponding author of this article, ORCID iD: 0000-0001-5306-3978

5. Thank you for stating the following financial disclosure: "This work was supported by the Natural Science Foundation of China under Grant#71704070（GD）; Outstanding Youth Fund of Gansu Province#20JR5RA206（GD）; Gansu Provincial Higher Education Research Project#2020A-058（GD）; Double First-class Key Scientific Research Project of Gansu Provincial Department of Education # GSSYLXM-06（GD）；Key scientific research project of Silk Road Economic Research Institute of Lanzhou University of Finance and Economics # JYYZ202102（GD）; Program of Lanzhou University of Finance and Economics under Grant#Lzufe2021B-002（GD）.The funders had no role in study design, data collection and analysis, decision to publish, or preparation of the manuscript."

We note that one or more of the authors is affiliated with the funding organization, indicating the funder may have had some role in the design, data collection, analysis or preparation of your manuscript for publication; in other words, the funder played an indirect role through the participation of the co-authors. If the funding organization did not play a role in the study design, data collection and analysis, decision to publish, or preparation of the manuscript and only provided financial support in the form of authors' salaries and/or research materials, please do the following:

a. Review your statements relating to the author contributions, and ensure you have specifically and accurately indicated the role(s) that these authors had in your study. These amendments should be made in the online form.

b. Confirm in your cover letter that you agree with the following statement, and we will change the online submission form on your behalf: 
“The funder provided support in the form of salaries for authors [insert relevant initials], but did not have any additional role in the study design, data collection and analysis, decision to publish, or preparation of the manuscript. The specific roles of these authors are articulated in the ‘author contributions’ section.

**Reply:** Thank you for your suggestion. This work was supported by the Natural Science Foundation of China under Grant#71704070(GD); Outstanding Youth Fund of Gansu Province#20JR5RA206(GD); Gansu Provincial Higher Education Research Project#2020A-058(GD); Double First-class Key Scientific Research Project of Gansu Provincial Department of Education # GSSYLXM-06(GD)；Key scientific research project of Silk Road Economic Research Institute of Lanzhou University of Finance and Economics # JYYZ202102(GD); Program of Lanzhou University of Finance and Economics under Grant#Lzufe2021B-002(GD).The recipients of the above funds are Guangyao Deng, the corresponding author of this article.

6. Your abstract cannot contain citations. Please only include citations in the body text of the manuscript, and ensure that they remain in ascending numerical order on first mention.

**Reply:** Thank you for your suggestion. Citations are no longer included in the abstract of revised manuscript.

7. Please upload a new copy of Figures 1-4 as the detail is not clear. Please follow the link for more information: https://blogs.plos.org/plos/2019/06/looking-good-tips-for-creating-your-plos-figures-graphics/" https://blogs.plos.org/plos/2019/06/looking-good-tips-for-creating-your-plos-figures-graphics/

**Reply:** Thank you for your suggestion. Figures have been converted to TIF and EPS format as requested and uploaded as Supporting Information files.

Additional Editor Comments:

As mentioned by the reviewers, this manuscript must have undergone significant revision. Please carefully revise this manuscript based on the comments of the reviewers and respond point by point.

Reply: Thank the editors and reviewers for the opportunity to revise this article.

Reviewers' comments:

Reviewer #1: This paper has discussed an interesting topic of matching between the transfer of embodied carbon emissions and value-added in China’s domestic setting. It is a research with important policy implications for carbon emission mitigation policy formulation. In addition to conventional input-output analysis, the authors went one step further to include a matching degree indicator to quantitatively compare the extent of mismatches between carbon and value-added trades.

**Reply:** Thank the reviewers for their valuable comments.

However, I feel that the rationale of matching degree indicator can be much improved. Specifically, the reasons of parameter setting and algorithm design are not well explained for eq (11) and eq (12). Although the algorithm is directly taken from Chen et al., it will much greatly improve the readability if the authors can explain why the authors have designed an algorithm as such and what are the physical implications of each equations and variables. I also feel that the last paragraph on page 19 and first paragraph on page 20 are essentially explanation for the algorithm design. They will be better associated with the explanation of eq (11) and (12) in the Methodology section.

**Reply:** Thank you for your suggestion. The design principle of Equations (11) - (12) is added in the revised manuscript, as follows:

When $\overline{DN}^{rs}>0$,it indicates that the net transfer out of embodied carbon from region *r* to region *s* is greater than 0, and both $\bar{w}^{rs}>0$and$\bar{w}^{rs}<0$ may exist. If $\overline{DN}^{rs}>0$ and $\bar{w}^{rs}>0$, it implies that the net transfer out of both embodied carbon and added value from region *r* to region *s* is greater than 0. The representative element $\frac{\overline{DN}^{rs}}{\bar{w}^{rs}}$in the matrix can be normalized to 0-1 by the function *f*(*y*).The larger the net transfer out of embodied carbon from region *r* to region *s*, and the smaller the net transfer out of added value, the closer $CI^{rs}$ is to 1. If $\overline{DN}^{rs}>0$ and $\bar{w}^{rs}<0$, the net transfer out of embodied carbon from region *r* to region *s* is greater than 0, but the net transfer out of added value is less than 0. In other words, the added value of region *r* is net transfer in from region *s*, which reflects the mismatch between the embodied carbon trade and the added-value trade. By Equation(11), the net transfer out of embodied carbon and the net transfer in of added value from region *r* to region *s*can be normalized to 0-1, and to distinguish from the first case, 1 is added on this basis.The larger the net transfer out of embodied carbon from region r to region s, and the higher the net transfer in of added value from region r to region s, the greater $CI^{rs}$ is greater than 1.It should be noted that when $\overline{DN}^{rs}=0$, the net embodied carbon transfer from area r to area s is less than 0, i.e., there is a net embodied carbon transfer from area r to area s. In addition, according to Equation (11),$CI^{rs}\neq CI^{sr}$, that is, the inequality of carbon emissions in the direction of net shift out and net shift in of each region is distinguished.

The corresponding modifications are now included in the revised manuscript. More details can be found in the revised manuscript.

In addition, as the authors have clearly declared on page 6 that provinces are aggregated into eight regions in accordance with document by the State Council, why are the heat maps in fig. 3 and fig. 4 not following the eight regions specification? Please state the reasoning of your choice.

**Reply:**Thank you for your suggestion. It may be that we have not described clearly. The results corresponding to the matching degree of embodied carbon trade and value-added trade in the eight regions of China are summarized in Table 6 and Table 7. Since the corresponding results have been listed in the table, the figures are not given.Fig. 3 and Fig. 4 are the results corresponding to the matching degree of embodied carbon trade and value-added trade in 31 provinces of China, and the paper chooses to display the results with graphs instead of tables.

Lastly, the authors seem to be stating how this research is different from other literatures in Further Discussion. However, I feel the differences should be integrated into literature review in Introduction. Discussion section should cover limitations and improvements instead.

**Reply:** Thank you for your suggestion. The original Discussion was deleted in the revised manuscript, and the discussion was rewritten in combination with the opinions of reviewers.

Reviewer #2: The article intends to estimate the embodied carbon trade and value-added trade within China, between its regions and provinces. Furthermore, the authors aim to reveal spatial disparities between these two flows in 2012 and 2015 with the help of MRIO-t. Unfortunately, though, several conceptual weaknesses of the analysis harm the robustness and the potential impacts on the field.

**Reply:** Thank the reviewers for their valuable comments.

First, the authors did not justify their starting point. The statement "In order to better clarify the carbon emission reduction responsibilities of provinces (regions), it is necessary to analyze the degree of matching between embodied carbon trade and value-added trade by combining the calculation results of them" on the 2nd page is not enough to introduce the research question. Is a high degree of matching something to achieve, or contrary, a low degree of matching? If an environmental policy targeted to equalize embodied carbon trade among regions, potential resource savings would be lost, for instance, in agricultural production (see work by Carole Dalin). Without any consideration in this regard, one cannot evaluate the study results, and so the authors themselves were unable to implement them.

**Reply:** Thank you for your suggestion. The revised manuscript further elaborated the research motivation of this paper, as follows:

The rewards brought by the value-added trade and the embodied carbon trade to different regions are not uniform.In most cases, the transfer in place of the value-added trade should pay an appropriate amount of remuneration for the products of the transfer out place of value-added trade. However, for the embodied carbon trade, there is a lack of corresponding ecological compensation mechanism. When a region transfers products with the high carbon emissions from other regions to replace the local production, the ecological environment of region is not damaged and the environmental cost is not paid. However, other regions need to bear the environmental pollution caused by the high carbon emissions, which leads to the so-called carbon leakage phenomenon(Deng and Xu,2017).In order to better clarify the carbon emission reduction responsibilities of provinces (regions), it is necessary to analyze the degree of matching between embodied carbon trade and value-added trade by combining the calculation results of them.

More details can be found in the revised manuscript.

Second, the population and the economic performance of the regions (provinces) should be controlled. The authors should have used per capita and GDP values of carbon emissions and trade instead of gross values.
**Reply:** As the reviewer pointed out, the carbon emissions and GDP (added value) are related to the population of each province (region). Zhang et al. (2018) referred to in this paper also analyzed the per capita pollutants and added value, but the total value is still used in the trade accounting. Chen et al. (2021) also used the total value when studying the matching degree of virtual land trade and virtual water trade. In fact, according to Equations (11) - (12), the per capita embodied carbon transfer out divided by the per capita added value transfer out is equal to the embodied carbon transfer out divided by the added value transfer out, and the trade matching degree $CI^{rs}$calculated by the two is consistent. In interpreting the results, the influence of population is also considered, as mentioned in the explanation of table 5. This is due to the fact that Guangdong Province is the most populous province in China, which needs to transfer a large number of agricultural products from other regions, while the higher labor input and larger value added coefficient in agriculture, together with the effect of value added trade in other industries, lead to a larger net value added transfer in the province.

Third, the results are poorly explained concerning their meaning (see the first issue) and socio-economic context. For example, the discussion on the geographical distance on the top of page 9 is not supported by any data; significant differences in 'net transfer out' in Table 5 comparing the two analyzed years are also not addressed. The reader is not provided with any information about the economic and trade structure at the regional level, the composition of trade and embodied carbon flows, etc. That is exactly why one writes academic articles; to reveal and explain a phenomenon.
Merits of the manuscript do not reach the publication criteria of the journal.

**Reply:** Thank you for your suggestion. Based on the classical trade gravity model, the trade volume between the two regions is directly proportional to the total economic volume and inversely proportional to the trade distance (Anderson and wincoop, 2003). The data in this paper also support the above conclusion. For example, the northern and eastern coastal areas are economically developed areas in China with high economic aggregate and adjacent geographical location, which results in relatively higher the volume of embodied carbon trade between these two areas. While, the economic aggregate of Northwest China is lower and the geographical location is far from the northern coastal area, which exhibits a relatively lower the embodied carbon trade between the two regions.

**References:**

Anderson J,Wincoop E (2003) Gravity with gravitas: a solution to the border puzzle. American Economic Review 93: 170-192.

In fact, according to the multi-regional input-output model constructed in this paper, one can get the embodied carbon trade volume and the value-added trade volume between various provinces and industries (The data for each year is a 930 × 31 order matrix, where, 30 and 31 represent the number of industries and provinces, respectively. In this study, the input-output tables are merged in industries according to the carbon emission data, which contain 42 industries in the CEADs database). Based on the calculated embodied carbon trade volume and value-added trade volume of each province and industry in 2012 and 2015, it can better explain the results in Table 5 from the perspective of trade structure. Due to the large amount of data, it only reports the main results in this paper and does not explain the results in Table 5 from the perspective of trade structure, which is the inadequacy of this paper.

Reviewer #3: This paper analyzed the embodied carbon and value-added in Chinese regional trade and constructed the associated matching index for year 2012 and 2015. Some interesting results have been presented in the paper. Below are the comments for the authors to improve the paper:

**Reply:** Thank the reviewers for their valuable comments.

[1.Introduction]

Literature review is not sufficient. The authors are suggested to have a separate section to review the studies done in the literature, highlight the gaps and unique contributions of this paper to the literature. Particularly, the relationship between embodied carbon emissions and embodied value added has been studied using the aggregate embodied intensity (AEI) concept proposed by Su and Ang (2017; Energy Economics 65, 137-147). The AEI indicator can be defined at the aggregation, regional, final demand, sectoral and transmission layers. See, for example, the multi-region AEI studies in Wang et al. (2020; Energy Economics 85, 104568), Su et al. (2021; Journal of Cleaner Production 313, 127894) and Wang et al. (2022; Technological Forecasting & Social Change 177, 121546) for China’s regions.

**Reply:** Thank you for your valuable advice. The references mentioned above have been added to the revised manuscript and the details can be found in the second paragraph of Introduction.

[2.Methods]

When formulating the national or multi-regional IO analysis, it is important to clarify the imports assumptions used in the analysis. See the first paper by Su and Ang (2013; Energy Policy 56, 83-87) discussing the impacts of imports assumptions in embodied emission estimates, and recent study by Su et al. (2022; Energy Economics 107, 105875) on the impacts of imports data treatments. The authors are suggested to add more discussions on imports assumptions as China usually compiled its IO tables using the competitive-imports assumption.

**Reply:** Thank you for your suggestion. The corresponding contents are added in the Discussion of this paper, as follows:

The input-output table of China (provinces) compiled by the national (provincial) bureau of statistics of China is usually the import competitive input-output table, while the multi-regional input-output table of China compiled in CEADs database is the import non-competitive input-output table. In the import competitive input-output table, the intermediate inputs consumed by each production departments do not distinguish between domestic production and import. It is assumed that they can be completely replaced, which means that the import column vector can be inserted directly in the third quadrant of the input-output table. While, the intermediate inputs in the noncompetitive input-output table are divided into two parts, namely the intermediate input of domestic production and the intermediate input of imports, which reflects the incomplete substitution of the two. In addition, the import column vector also exists in the final demand part. Since the import non-competitive input-output table is adopted in this paper, it is not necessary to mention the impact of imported products. The related literatures on the import competitive and non-competitive input-output tables can be found in Su and Ang, and Su et al.

More details can be found in the revised manuscript.

**References:**

Su B, Ang B W (2013) Input–output analysis of CO2 emissions embodied in trade: competitive versus non-competitive imports. Energ Policy 56: 83-87.

Su B, Ang B W, Sun Y F (2022) Input-output analysis of embodied emissions: Impacts of imports data treatment on emission drivers. Energy Economics 107: 105875.

When using only China’s MRIO table, the embodied flows only capture China’s interregional feedback effects, not including the international feedback effect (Su and Ang, 2011; Ecological Economics 71, 42-53). See, for example, the hybrid model in Su and Ang (2014; Applied Energy 114, 377-384) and embedded model in Su et al. (2021; Journal of Cleaner Production 313, 127894). The calculation of the matching index could be different when international feedback effects are captured. The authors are suggested to add more discussions in this sector and the limitations (or future research) of the study in the last section.

**Reply:** Thank you for your suggestion. The corresponding contents are added in the Discussion of this paper, as follows:

The present paper is mainly concerned with the matching degree of embodied carbon trade and value-added trade among Chinese provinces (regions). Only the matching degree of domestic trade is considered, and the matching degree of international trade is not involved. Furthermore, the impact of international trade is not embedded in the consideration of domestic trade (Su and Ang, 2011;Su and Ang,2014;Su et al. 2021). If the domestic trade and international trade are considered comprehensively, the matching degree between the embodied carbon trade and value-added trade of each province (region) may be different from the calculation results of this paper.

More details can be found in the revised manuscript.

**References:**

Su B, Ang B W, Liu Y (2021) Multi-region input-output analysis of embodied emissions and intensities: Spatial aggregation by linking regional and global datasets. J Clean Prod 313: 127894.

Su B, Ang B W (2011) Multi-region input–output analysis of CO2 emissions embodied in trade: the feedback effects. Ecol Econ 71: 42-53.

Su B, Ang B W(2014) Input–output analysis of CO2 emissions embodied in trade: a multi-region model for China. Appl Energ 114: 377-384.

Eq. (11): How to do the calculation when W^rs=0 or W^rs becomes very small, e.g. 10^(-30)? Robustness of the matching index formulation should be discussed.

**Reply:** Thank you for your suggestion. The third case of Equation (11) is modified in the revised manuscript. When W^rs=0,CI^rs=0 is defined directly, which means the matching degree is 0.This is not the case in the empirical results of this paper, and thank the reviewers for pointing out the shortcomings of this paper.

Data: CEADS only compiled China’s regional carbon emissions for 30 regions, not including Tibet (not “Xizang” in the table). Where did the authors get the energy/emissions data for Tibet in 2012 and 2015?

**Reply:** Thank you for your suggestion. Since China's multi-regional input-output table in CEADs database includes Tibet, but lacks its energy and carbon emission data of various industries, this research assumes that the direct carbon emission coefficient of various industries in Tibet is equal to the average value of other 30 provinces, which is used to estimate the carbon emission of Tibet. In addition, because Tibet has less trade links with other provinces, there is less embodied carbon trade with other provinces. Based on the above viewpoint, the treatment of carbon emissions in Tibet will not cause a large deviation to the results.

[3.Results and Discussions]

The authors presented the results in the eight aggregated regions, which losing some critical information at the detailed provincial level. The authors are suggested to include all the 31 regions’ results (e.g. embodied emissions and value added in interregional trade) in the discussions or at least provide the detailed results in table in the Appendix.

**Reply:** Thank you for your suggestion. Because the result of embodied carbon trade or value-added trade among the 31 provinces is a 31× 31 order matrix, it is not convenient to display with figures or tables in the paper. Therefore, only the total transfer out volume and transfer in volume of each province are listed, and the detailed results can be found in Table 3 and Table 5.

In addition, the following contents are supplemented in the Discussion of this paper. It should be pointed out that, according to the multi-regional input-output model constructed in this paper, one can get the embodied carbon trade volume and the value-added trade volume between various provinces and industries (The data for each year is a 930 × 31 order matrix, where, 30 and 31 represent the number of industries and provinces, respectively. In this study, the input-output tables are merged in industries according to the carbon emission data, which contain 42 industries in the CEADs database). Based on the calculated embodied carbon trade volume and value-added trade volume of each province and industry in 2012 and 2015, it can better explain the results in Table 5 from the perspective of trade structure. Due to the large amount of data, it only reports the main results in this paper and does not explain the results in Table 5 from the perspective of trade structure, which is the inadequacy of this paper.

Table 5: Are the embodied value added figures given for 2012 and 2015 comparable if not giving them in the fixed year price?

**Reply:** Thank you for your suggestion. In Data of this paper, it is supplemented that this study takes 2012 as the base period and deflates the data related to prices in 2015.

The authors are also suggested co compare their results (e.g. embodied emissions) with those reported in the literature using the similar MRIO dataset for China, including the regional AEI studies for China.

**Reply:** Thank you for your suggestion. According to the suggestions of the first reviewer, the literature comparison is directly placed in Introduction of this paper, and Discussion only retains the limitations of this paper and possible further improvements.

[Conclusions and implications]

The authors are suggested to add more discussions on the findings on the mismatching index, which is the major contribution in the paper, for policy makings. What are the possible ways to improve the inequality or mismatching for future regional developments?

**Reply:** Thank you for your suggestion. According to the second suggestion of editor (Please note that PLOS ONE is designed to communicate original research and research methods, and we do not consider policy papers. As such, we ask you to remove policy discussion and recommendations made in your Conclusions section and ensure that statements made in your conclusions are supported by the data in your article), the Policy discussion and recommendations in the original manuscript has been removed.

In Introduction, it gives a further explanation of the motivation of this study. It should be noted that the rewards brought by the value-added trade and the embodied carbon trade to different regions are not uniform. In most cases, the transfer in place of the value-added trade should pay an appropriate amount of remuneration for the products of the transfer out place of value-added trade. However, for the embodied carbon trade, there is a lack of corresponding ecological compensation mechanism. When a region transfers products with the high carbon emissions from other regions to replace the local production, the ecological environment of region is not damaged and the environmental cost is not paid. However, other regions need to bear the environmental pollution caused by the high carbon emissions, which leads to the so-called carbon leakage phenomenon. In order to better clarify the carbon emission reduction responsibilities of provinces (regions), it is necessary to analyze the degree of matching between embodied carbon trade and value-added trade by combining the calculation results of them.

The authors may consider using the structural decomposition analysis (SDA) to investigate the drivers of the matching index in future research. See the review of SDA studies on energy and emissions in Su and Ang (2012; Energy Economics 34 (1), 177-188) and Wang et al. (2017; Energy Policy 107, 585-599).

**Reply:** Thank you for your suggestion. The corresponding contents are added in the Discussion of this paper, as follows:

Moreover, it is also the further research direction of this paper to study the driving factors of the matching degree of embodied carbon trade and value-added trade among Chinese provinces (regions) combining with the structural decomposition analysis(Su and Ang,2012;Wang et al.,2017).

More details can be found in the revised manuscript.

**References:**

Su B, Ang B W(2012) Structural decomposition analysis applied to energy and emissions: some methodological developments. Energy Economics 34(1): 177-188.

Wang H, Ang B W, Su B(2017) Assessing drivers of economy-wide energy use and emissions: IDA versus SDA. Energ Policy 107: 585-599.
